# Mediating Effects of Emotional Support Reception and Provision on the Relationship between Group Interaction and Psychological Well-Being: A Study of Young Patients

**DOI:** 10.3390/ijerph182212110

**Published:** 2021-11-18

**Authors:** Steven Sek-yum Ngai, Chau-Kiu Cheung, Jianhong Mo, Spencer Yu-hong Chau, Elly Nga-hin Yu, Lin Wang, Hon-yin Tang

**Affiliations:** 1 Department of Social Work, The Chinese University of Hong Kong, Hong Kong, China; lannymo@cuhk.edu.hk (J.M.); chauy@usc.edu (S.Y.-h.C.); ellynh@cuhk.edu.hk (E.N.-h.Y.); linwang@cuhk.edu.hk (L.W.); hytang@cuhk.edu.hk (H.-y.T.); 2 Department of Social and Behavioral Sciences, City University of Hong Kong, Hong Kong, China; ssjacky@cityu.edu.hk

**Keywords:** mutual aid group, emotional support reception/provision, psychological well-being, young patients, chronic health conditions

## Abstract

While it is well-established that mutual aid groups are effective in the psychological rehabilitation of vulnerable individuals, few studies have thoroughly investigated the dynamic mechanism of how psychological well-being improves through mutual aid groups of young patients with chronic health conditions. In connection with several existing theories (i.e., the helper therapy principle, equity theory, the norm of reciprocity, and the concept of communal relationships), this study aims to: (1) evaluate whether emotional support exchanges (i.e., emotional support reception and provision) mediate the relationship between group interaction and psychological well-being; and (2) compare three potential underlying mechanisms—the mediating role of emotional support provision, equitable reciprocity (i.e., a balance of receiving and providing emotional support, where no party over-benefits or under-benefits), and sequential reciprocity (i.e., repaying the helper or a third party in the future after receiving help)—through a path analysis model. A stratified random sampling procedure with chronic health conditions as the stratifying criterion was used to recruit 391 individuals aged 12–45 years from mutual aid groups in Hong Kong, who completed both the baseline and follow-up surveys over a 12-month interval. The results of the path model revealed significant mediating roles of emotional support provision and sequential reciprocity, not equitable reciprocity. The present study offers theoretical and practical implications for promoting the psychological well-being of young patients with chronic health conditions.

## 1. Introduction

Chronic health conditions are illnesses persisting for at least one year (such as cancer, asthma, diabetes, and heart disease) that restrict daily activities and require ongoing treatment [1]. In addition to the increasing prevalence of chronic health conditions recorded worldwide [2], the early onset of chronic health conditions has become another common trend that no longer concerns only the older population. This emerging trend is attributable to widespread unhealthy lifestyles, such as poor dietary habits and physical inactivity [3]. The pervasiveness of chronic health conditions in younger age groups also grows with medical advancement, as more children are able to survive conditions that would have been fatal in the past [4]. In Hong Kong, approximately 12.6% of people younger than 45 years have chronic health conditions [5]. Besides enduring various challenging physical obstacles—including demanding treatment regimens, treatment side effects, and physical sequelae concomitant with their chronic conditions—these young patients with chronic health conditions (PCHC) need to manage their emotional reactions to stressful events and adjust to physical and social limitations [6]. Furthermore, young PCHC must also face the challenge of handling the illness and treatments while simultaneously integrating an emerging personal identity with future educational and occupational opportunities [7]. In addition to physical limitations, young PCHC tend to avoid interacting with and developing interpersonal relationships with others, due to illness-related shame and the sense of social misunderstanding and rejection; thus, they report worse mental health status [8]. Previous studies have shown that young PCHC face a greater risk of psychiatric problems, such as symptoms of depression and lower psychological well-being [9].

An extensive amount of literature has indicated that young PCHC can benefit from mutual aid groups [10]. Mutual aid groups are groups formed by people with similar problems and needs, who come together for mutual assistance based on their own resources and experiences [11,12]. The mutual aid group setting enables members to interact with others similar to them and to enrich their social networks, thus fostering the sharing and exchanging of information, experience, and emotions [13]. Participation in mutual aid groups has been beneficial in relation to many rehabilitation outcomes, including improved mental health status [14], enhanced illness management [15], reduced symptoms of depression and anxiety [16], and better psychological well-being [17].

While numerous studies have focused on the effectiveness of mutual aid groups, they have rarely described the underlying mechanism of how group processes (such as group interaction and the accompanying emotional support among members) enable such changes. To address this research gap, the current study aims to examine the underlying mechanism enhancing young PCHC’s rehabilitation outcomes, especially psychological well-being in mutual aid groups.

### 1.1. Importance of Psychological Well-Being for Young PCHC’s Rehabilitation

Psychological well-being refers to the extent to which a person feels that their life is going well and is meaningful [18,19]. Two complementary traditions of psychological well-being have been developed: the hedonistic tradition equates psychological well-being with happiness and satisfaction derived from a global evaluation of life (e.g., life satisfaction), while the eudaemonic tradition centers on living up to one’s full potential in a meaningful way (e.g., pursuing life purpose and personal growth). To maintain a comprehensive view, the current study conceives of psychological well-being as a multidimensional construct that consists of three aspects: life satisfaction, purpose in life, and personal growth. Life satisfaction concerns one’s cognitive evaluation of one’s life as a whole [19]; purpose in life broadly refers to one’s ability to recognize one’s meaning in life, to have goals and directions, and to hold beliefs that provide a purpose to live [20]; and personal growth implies one’s ability to recognize one’s own growth, continuous development, and potential, as well as to make progress and improvements that align with one’s self-knowledge [20]. Previous studies on PCHC have reported that psychological well-being is connected to health maintenance, health outcomes, disease or symptom control, survival, and longevity [21,22], while poorer psychological well-being is associated with lower treatment adherence, complications, and greater mortality for PCHC [23]. Given the positive associations between psychological well-being and health outcomes, it is a critical indicator of wellness for young PCHC.

### 1.2. Effects of Group Interaction on Emotional Support Exchanges

Group interaction denotes communications among group participants, including their initiatives, attentiveness, and responsiveness to each other within the group [24,25]. Group interactions can help boost emotional support exchanges in mutual aid group settings. For instance, interacting with others who have endured similar experiences and difficulties increase feelings of social understanding and acceptance [26]. Such interactions also further cultivate a sense of belonging and community, which strengthens the “in the same boat” perception and the trust within mutual aid groups such that group members perceive each other as peers who are trustworthy, thus facilitating and fostering richer exchanges of emotional support [13,27].

### 1.3. Emotional Support Reception and Provision as Mediators

Mutual aid is a kind of interactive support that occurs between people with similar concerns or problems, such as PCHC [28]. It comes in various forms, but most commonly, it consists of instrumental support (e.g., tangible service), informational support (e.g., advice), and emotional support (e.g., empathy and love) [29]. Among these, the current study chose to focus primarily on emotional support, as past literature suggests its relative availability among PCHC and its positive impacts on health outcomes and on well-being [30,31]. There are two key operative processes in mutual aid groups—receiving emotional support and providing emotional support—which constitute a reciprocal process [32]. According to Brown et al. (2014) [29], emotional support reception refers to being heard, acknowledged, and respected by fellow group members, while emotional support provision refers to listening attentively as well as expressing concerns and encouragement in support of the emotional needs of fellow group members.

Given the effect of group interaction on emotional support and the impact of emotional support on young PCHC’s psychological well-being, the current study seeks to examine how emotional support reception and provision positively mediates the relationship between group interaction and young PCHC’s psychological well-being—that is, how group interaction may boost emotional support reception and provision, which in turn improves young PCHC’s psychological well-being. According to previous literature, these effects are viable with three different underlying mechanisms: the mediating role of emotional support provision, equitable reciprocity, and sequential reciprocity, which are explicated as follows.

### 1.4. Benefits of Providing Emotional Support, Based on the Helper Therapy Principle

The helper therapy principle, often used to delineate the benefits of mutual aid groups [29,33], theorizes that those who also provide support benefit more than those who merely receive it. While studies on receiving support have identified inconclusive effects [34], studies on providing support consistently exhibit that support providers can benefit in several indirect or unintended ways. For instance, by extending help, a person has improved their self-image and well-being as a result of the act of doing something deemed worthwhile [35], and they can also inadvertently encourage themselves when attempting to encourage other group members [33]. Furthermore, as a by-product, support providers lighten their stress when they shift their focus away from their own suffering as they attempt to help others, while they also simultaneously gain a sense of purpose from their acts of helping [29]. In addition, past studies have recorded improvements in psychological well-being (purpose in life, personal growth, and life satisfaction) alongside other health outcomes as a result of providing emotional support [35,36]. Given these well-documented benefits, a set of hypotheses for this study reads as follows:

**Hypothesis** **1.1** (**H1.1**): *Group interaction increases emotional support provision in mutual aid groups, thereby positively affecting young PCHC’s purpose in life*.

**Hypothesis** **1.2** (**H1.2**): *Group interaction increases emotional support provision in mutual aid groups, thereby positively affecting young PCHC’s personal growth*.

**Hypothesis** **1.3** (**H1.3**): *Group interaction increases emotional support provision in mutual aid groups, thereby positively affecting young PCHC’s life satisfaction*.

### 1.5. Two Competing Theories: Equitable Reciprocity and Sequential Reciprocity

Although solely providing emotional support may improve young PCHC’s psychological well-being, previous literature has also examined how the processes of receiving and providing emotional support unfold in mutual aid groups. Two mechanisms, equitable reciprocity and sequential reciprocity, are relevant in this context.

#### 1.5.1. Equitable Reciprocity

In their attempts to outline the reciprocal mechanism in mutual aid groups, many studies have turned to equity theory. This theory posits that individuals who are aware of their participation in inequitable situations experience emotional distress, which they will try to eliminate by attempting to restore equity via an act of reciprocation [37,38]. In other words, individuals are most comfortable when they are participating in equitable relationships characterized by a balance of receiving and providing emotional support, where no party over-benefits or under-benefits.

An impressive body of literature across different contexts has indicated positive outcomes resulting from reciprocal support, based on propositions from equity theory. For example, in a study conducted by Pandit and Nakagawa (2021) [37], receiving additional emotional support (i.e., increased inequity) did not significantly reduce one’s depression if one did not provide additional emotional support (i.e., no balanced exchange), suggesting an interaction effect between emotional support reception and provision. This outcome was also in line with Maton’s (1988) bidirectional support hypothesis [39], wherein bidirectional supporters (high support reception and provision) experienced more positive well-being compared to unidirectional supporters (high in either support reception or provision only) and to non-supporters. More explicitly, individuals who managed to engage in balanced and equitable relationships tended to fare better in terms of well-being than those who over-reciprocate or under-reciprocate [40].

#### 1.5.2. Sequential Reciprocity

Although equity theory is plausible for understanding the reciprocal dynamics of helping relationships, its emphasis on balanced exchanges does not take into consideration more complex variations. In fact, studies on whether interpersonal relationships follow equity theory are inconclusive at best [41]. Thus, the current study considers an alternative form of reciprocity—sequential reciprocity—that is rooted in the norm of reciprocity characterized by communal relationships.

According to Gouldner (1960) [42], the norm of reciprocity follows two simple demands: that recipients should return help to those who helped them, and that they should not inflict harm on their helpers. In this norm, the demand for “balanced” exchanges is unnecessary; contrary to equity theory, which posits that no one should under-benefit or over-benefit in an exchange, the norm of reciprocity suggests that individuals will only resist over-benefitting in a relationship [41]. Based on this logic, individuals who receive support will feel morally obliged to return the favor in order to avoid over-benefitting. However, when it is their turn to provide help, they will not react negatively if they receive no reciprocation, contradicting Maton’s [39] bidirectional argument that one requires both receiving and providing support to reach the most optimal outcome.

Moreover, according to Roberts et al. (1999) [32], mutual aid groups denote a system of communal relationships, where participants tend to develop supportive ties with each other over time. This type of relationship is often missing in equity theory. According to Clark and Mills (1979) [43], communal relationships refer to tight-knit communities, where each member is concerned about the general well-being of the group. Rather than focusing on maintaining balanced relationships, those participating in communal relationships proactively provide aid out of general concern or in response to members’ needs, ignoring rules that stipulate the return of equivalent support [43]. Communal relationships are generally tolerant of asymmetries and do not demand immediate reciprocation [43]. In fact, reciprocations in networks with strong ties can be time-delayed and indirect, meaning that members can return the favor months or years after receiving the aid (such as in intergenerational reciprocity) and that the favor can be returned indirectly—i.e., to other group members, rather than directly to the benefactor only [41].

Given the above distinction between equitable reciprocity and sequential reciprocity, the following two sets of competing hypotheses are proposed:

#### Equitable Reciprocity

**Hypothesis** **2.1** (**H2.1**): *Group interaction increases equitable reciprocity (e.g., high levels of both emotional support provision and reception) in mutual aid groups, thereby positively affecting young PCHC’s purpose in life*.

**Hypothesis** **2.2** (**H2.2**): *Group interaction increases equitable reciprocity in mutual aid groups, thereby positively affecting young PCHC’s personal growth*.

**Hypothesis** **2.3** (**H2.3**): *Group interaction increases equitable reciprocity in mutual aid groups, thereby positively affecting young PCHC’s life satisfaction*.

#### Sequential Reciprocity

**Hypothesis** **3.1** (**H3.1**): *Group interaction increases sequential reciprocity (i.e., increases emotional support reception and then emotional support provision) in mutual aid groups, thereby positively affecting young PCHC’s purpose in life*.

**Hypothesis** **3.2** (**H3.2**): *Group interaction increases sequential reciprocity in mutual aid groups, thereby positively affecting young PCHC’s personal growth*.

**Hypothesis** **3.3** (**H3.3**): *Group interaction increases sequential reciprocity in mutual aid groups, thereby positively affecting young PCHC’s life satisfaction*.

## 2. Methods

### 2.1. Procedure

This study adopted a panel study design to collect two waves of data examining relationships among group interaction, emotional support reception and provision, and psychological well-being of young PCHC, with an average 12-month interval between the baseline and follow-up surveys. Participants completed the baseline survey between 2017 and 2018, and the follow-up survey between 2018 and 2019. A full list of mutual aid groups for PCHC was made available through the aid of a major non-governmental organization that focuses on supporting persons with disabilities or health challenges in Hong Kong. A stratified random sampling method with chronic health conditions as the stratifying criterion was used, in which the mutual aid groups for PCHC were further divided into ten prevalent chronic health conditions: asthma, heart disease, diabetes, rheumatic diseases, neurological diseases, hematologic diseases, cancer, rare diseases, eczema, and mental illnesses. The sampling selected 50 mutual aid groups in Hong Kong, with 5 groups from each of these chronic health conditions. Trained interviewers conducted the surveys through face-to-face interviews with the participants at the service centers of the mutual aid groups.

All members aged 12–45 years of the selected mutual aid groups participated in this study. The participants provided informed consent as to the objectives and data collection procedure of this study. For those younger than 18 years, their parents provided informed consent as well. An ethical review committee evaluated and approved this method prior to administration. A total of 497 young PCHC participated in the baseline survey, of whom 391 participated in the follow-up survey. Thus, the retention rate was high (approximately 80%). Moreover, the attrition was unrelated to the variables involved in the study, according to a logistic regression analysis of follow-up responses versus attrition (Cox and Snell *R*^2^ = 0.011).

### 2.2. Participants

Of the final 391 participants involved in both the baseline and follow-up surveys, slightly more than half (55.1%, see Table 1) were female. The mean age of participants was 31.0 years (SD = 7.7). Most of the participants (75.9%) had a monthly family income of HKD 30,000–39,999 (USD 3871–5160) or below. In terms of education, 96.5% of participants have completed secondary school (grades 7–12) or above. The most frequently reported chronic health condition among the participants was mental illnesses (23.0%), followed by eczema (18.8%), rheumatic diseases (17.5%), neurological diseases (10.7%), hematologic diseases (6.5%), diabetes (5.5%), cancer (5.2%), asthma (5.0%), rare diseases (4.7%), and heart disease (3.1%).

Participants reported that the average frequency of mutual aid group activities was 1.8 times (SD = 3.7), with an average duration of 1.5 h (SD = 2.5) per group activity, during the past six months. The average number of members was 13.2 (SD = 8.0) in a mutual aid group, with 5.0 (SD = 3.9) of them being core members serving in a leadership or coordination role. On average, a mutual aid group had 1.6 (SD = 2.0) professional facilitators such as social workers or allied health workers and 0.9 (SD = 1.9) nonprofessional facilitators who were ex-patients who had experienced chronic health conditions with good current management of their conditions.

### 2.3. Measurement

The measures used in the current study were adapted from validated scales found in previous research that was conducted in the local or overseas contexts, with proven high internal consistency [29,44,45,46,47,48]. All measures used in the current study were scored on a five-point rating scale, ranging from “never or rarely” to “very often”. To facilitate data interpretation and comparison, all scores lay on a 0–100 scale [49], with higher scores indicating higher levels of performance on each measure. Additionally, different time frames were applied to different sections of the questionnaire. For instance, participants were asked about the “past six months” when answering questions regarding group interaction as well as emotional support reception and provision, while they were asked about the “past month” for questions on psychological well-being.

#### 2.3.1. Group Interaction

Group interaction consists of communications among group participants, including their initiatives, attentiveness, and responsiveness to each other within the group [25,26]. The group interaction measure contained four items that focused on the frequency of certain group communication-related behaviors in the past six months [46,47]. Sample items include “Group members encourage each other to express their opinions” and “Group members take the initiative to make comments.” The measure of group interaction was administered at baseline. Its Cronbach’s alpha was 0.961, indicating good reliability.

#### 2.3.2. Emotional Support

The concept of emotional support consists of exchanges of positive feedback during communication—such as care, encouragement, affirmations, and praise—received from group members or provided to group members. The emotional support measure contained a total of six items, with three items for emotional support reception and three for emotional support provision [29]. This measure asked participants to report behaviors indicating emotional support reception from or provision to group members in the previous six months. Sample items of emotional support reception include “How often did group members listen carefully when you talked?” and “How often did group members show interest in hearing your ideas and opinions?” Sample items of emotional support provision include “How often did you express concern for a group member?” and “How often did you provide emotional support to a group member?” Emotional support reception was measured at baseline, while emotional support provision was measured at follow-up; the corresponding Cronbach’s alpha was 0.971 and 0.964, respectively, showing good reliability.

#### 2.3.3. Psychological Well-Being

Psychological well-being denotes the extent to which a person feels that their life is going well and is meaningful [19,20] which is indicated in this study by three distinctive components: purpose in life, personal growth, and life satisfaction. The measure of purpose in life included four items, asking participants’ subjective judgment of their life significance as well as their abilities to make sense of life and to find value in life over the previous month [48]. Sample items include “How often did your life have a clear sense of purpose?” and “How often did you find your life purpose satisfied?” The measure of personal growth contained four items [44], which asked participants to indicate their continuous development in reaching their full potential in the previous month. Sample items include “How often did you make yourself a better person?” and “How often did you keep trying at something until you succeeded?” The measure of life satisfaction contained four items [45] assessing participants’ personal judgment toward their quality of life in the previous month [21]. Sample items include “How often did you find that your life and your philosophy matched well?” and “How often did you find you were satisfied with your life?” The measures of psychological well-being were administered at both baseline and follow-up. Cronbach’s alpha for the purpose in life, personal growth, and life satisfaction measures at baseline was 0.870, 0.676, and 0.854, respectively; Cronbach’s alpha for the purpose in life, personal growth, and life satisfaction measures at follow-up was 0.875, 0.646, and 0.844, respectively. Thus, reliability for the measures on purpose in life and life satisfaction was good, and reliability for the personal growth measure was acceptable.

### 2.4. Data Analysis Plan

**Figure 1:**
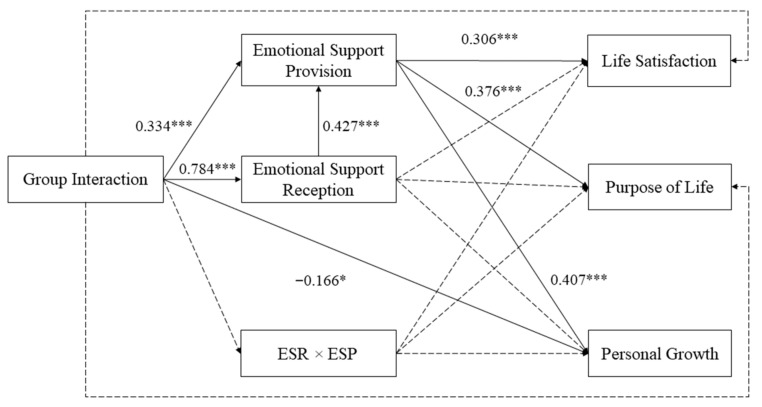
Standardized solutions for the integrated model with the control for background and baseline psychological well-being variables. Full lines represent significant relationships. Dotted lines represent non-significant relationships. ESR × ESP = emotional support reception (baseline) × emotional support provision (follow-up). * *p* < 0.05. *** *p* < 0.001.

**Figure 2:**
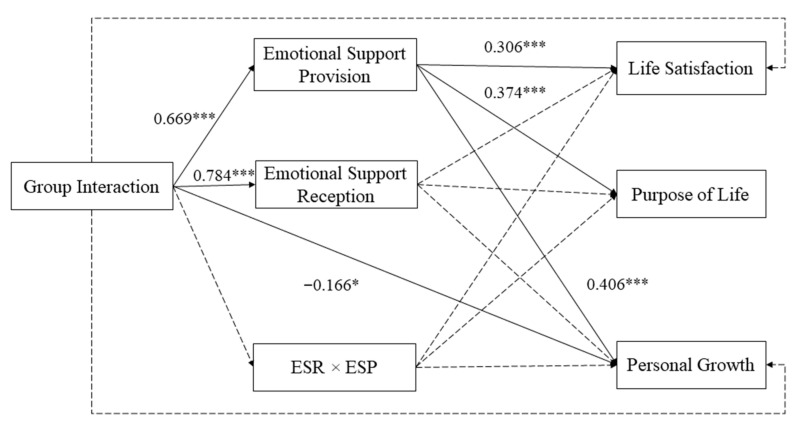
Standardized solutions for the equitable reciprocity model with the control for background and baseline psychological well-being variables. Full lines represent significant relationships. Dotted lines represent non-significant relationships. ESR × ESP = emotional support reception (baseline) × emotional support provision (follow-up). * *p* < 0.05. *** *p* < 0.001.

**Figure 3:**
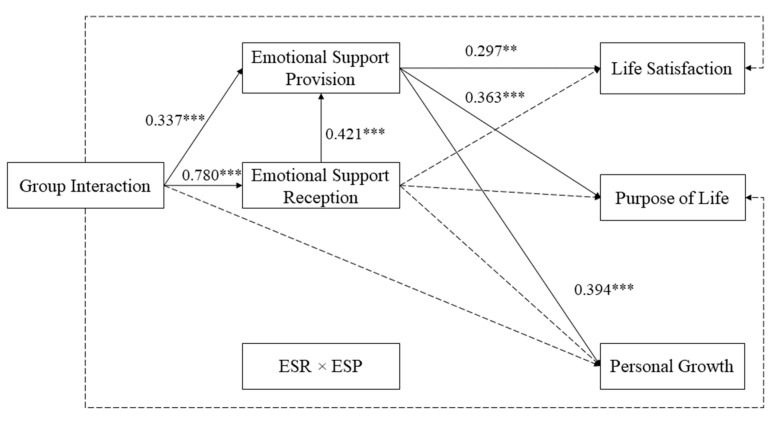
Standardized solutions for the sequential reciprocity model with the control for background and baseline psychological well-being variables. Full lines represent significant relationships. Dotted lines represent non-significant relationships. ESR × ESP = emotional support reception (baseline) × emotional support provision (follow-up). ** *p* < 0.01. *** *p* < 0.001.

Descriptive and preliminary analyses, including means, standard deviations, and Pearson’s correlations, were conducted in SPSS 24.0 (IBM Corp., Armonk, NY, USA). First of all, to test the coexistence of equitable and sequential reciprocity hypotheses, an integrated path model was assessed using Mplus 8.0 software (Muthen & Muthen, Los Angeles, CA, USA) (see Figure 1) [50]. This model included an interaction term created by multiplying the standard score of emotional support reception at baseline by the standard score of emotional support provision at follow-up (to represent the hypotheses related to equitable reciprocity [32,51]) and a sequential mediation of emotional support reception and emotional support provision (to represent the hypotheses related to sequential reciprocity [52]) in order to examine their effects on the relationship between group interaction and psychological well-being. An equitable reciprocity model then constrained the path from emotional support reception to emotional support provision in the integrated model to be zero (see Figure 2) in order to test the hypotheses related to equitable reciprocity alone. Alternatively, a sequential reciprocity model was constructed based on the integrated model by constraining (a) the path from group interaction to the interaction term, as well as (b) the paths from the interaction term to psychological well-being variables to be zero (see Figure 3) in order to test the hypotheses related to sequential reciprocity alone. Thus, both the sequential reciprocity and equitable reciprocity models were nested in the integrated model for meaningful model comparisons. All the modeling applied the robust maximum likelihood estimator. Background factors (age, gender, family income, educational level, and chronic health condition) and outcomes (purpose in life, personal growth, and life satisfaction) at baseline were covariates. Recommended cut-off values for a good model fit are comparative fit index (CFI) greater than 0.95, as well as root mean square error of approximation (RMSEA) and standardized root mean square residual (SRMR) lower than 0.08 [53,54,55].

## 3. Results

### 3.1. Descriptive Statistics and Preliminary Analyses

Table 2 shows descriptive statistics (i.e., mean and SD) of the main variables, along with a correlation matrix summarizing bivariate correlations. As shown, all the variables were significantly correlated with each other in expected patterns. Group interaction (measured at baseline) was positively associated with emotional support reception (measured at baseline) as well as emotional support provision (measured at follow-up) and the three aspects of psychological well-being—purpose in life, life satisfaction, and personal growth (measured at follow-up). Emotional support reception (baseline) and emotional support provision (follow-up) were also positively associated with the three variables of psychological well-being (follow-up). The correlation coefficients ranged from weak to strong.

### 3.2. Path Analysis

Table 3 shows the absolute fit indices of the three tested models. According to the cut-off criteria we adopted, the integrated model and the sequential reciprocity model both had a satisfying fit (the integrated model: *χ*^2^ = 3.432, *df* = 1, *χ*^2^/*df* = 3.432, CFI = 0.999, RMSEA = 0.079, SRMR = 0.008; the sequential reciprocity model: *χ*^2^ = 47.813, *df* = 22, *χ*^2^/*df* = 2.173, CFI = 0.984, RMSEA = 0.055, SRMR = 0.053), while the equitable reciprocity model did not (the equitable reciprocity model: *χ*^2^ = 27.784, *df* = 2, *χ*^2^/*df* = 13.892, CFI = 0.984, RMSEA = 0.182, SRMR = 0.019). However, the path coefficients from the interaction term to dependent variables were all non-significant, which suggested that the equitable reciprocity hypotheses were not supported. Moreover, based on the parsimony principle, the sequential reciprocity model should be selected as our final model because these two models fit equally, but the sequential reciprocity model has a much larger degree of freedom (*df* = 22) than that of the integrated model (*df* = 1).

According to the sequential reciprocity model, the total effects of group interaction on purpose in life (*β* = 0.130, *p* < 0.05; see Table 4) and on life satisfaction (*β* = 0.100, *p* < 0.05) were significant; its effect on personal growth was also marginally significant (*β* = 0.085, *p* = 0.059). These total effects were decomposed into direct effects and indirect effects (see Figure 1). Specifically, the direct effect of group interaction on purpose in life was not significant (*β* = −0.026, *p* > 0.05; see Table 5); similarly, the direct effects of group interaction on life satisfaction and personal growth were also not significant (*β* = −0.083, *p* > 0.05 for life satisfaction; *β* = −0.137, *p* > 0.05 for personal growth).

The first set of hypotheses, concerning the helper therapy principle, was evaluated by the simple mediation effects of emotional support provision in the sequential reciprocity model. The indirect effects of group interaction via emotional support provision on purpose in life (*β* = 0.122, *p* < 0.01; see Table 4), on personal growth (*β* = 0.133, *p* < 0.01), and on life satisfaction (*β* = 0.100, *p* < 0.01) were all significant, thus supporting Hypotheses 1.1, 1.2, and 1.3. However, the simple mediation effects of emotional support reception on the relationships between group interaction and the three psychological well-being variables were not significant.

The second set of hypotheses, concerning equitable reciprocity, was evaluated by the equitable reciprocity model (see Figure 2). The corresponding results showed that the associations of the interaction term with psychological well-being variables were not significant (*β* = −0.057, *p* > 0.05 for purpose in life; *β* = −0.065, *p* > 0.05 for personal growth; *β* = −0.041, *p* > 0.05 for life satisfaction; see Table 5). Therefore, Hypotheses 2.1, 2.2, and 2.3 were not supported.

The third set of hypotheses, concerning sequential reciprocity, was tested by the sequential mediation effect of emotional support in the sequential reciprocity model (see Figure 3). As hypothesized, group interaction was indirectly associated with purpose in life (*β* = 0.119, *p* < 0.001), personal growth (*β* = 0.129, *p* < 0.001), and life satisfaction (*β* = 0.097, *p* < 0.01), first through emotional support reception and then through emotional support provision. Specifically, group interaction led to an increased level of emotional support reception, which in turn resulted in higher levels of emotional support provision, thereby ultimately facilitating various outcomes of psychological well-being. The overall path model accounted for 41.7% of the variance in purpose in life, 47.0% of the variance in personal growth, and 43.7% of the variance in life satisfaction. In short, the sequential mediation effects of emotional support reception and emotional support provision were significant on the relationships between group interaction and the psychological well-being variables; therefore, Hypotheses 3.1, 3.2, and 3.3 were supported.

In terms of the covariates (see Table 5), among the chronic health conditions, asthma and diabetes were negatively predictive of purpose in life; asthma, eczema, and hematologic diseases were negatively predictive of life satisfaction; mental illnesses and eczema were negatively predictive of personal growth. The direct associations between other chronic health conditions and psychological well-being were non-significant.

## 4. Discussion

Despite being a widely-acknowledged approach for rehabilitation, mutual aid groups remain poorly understood in terms of the specific mechanisms through which they facilitate psychological well-being. Drawing on a sample of young PCHC in Hong Kong, the present study aimed to examine the associations between group interaction and different dimensions of psychological well-being, including purpose in life, personal growth, and life satisfaction, with particular focus on the mediation effects of emotional support reception, emotional support provision, and their interplay on these relationships. Three major findings were revealed. First, emotional support provision plays a mediating role between group interaction and psychological well-being variables. Second, the interaction term (i.e., emotional support reception × emotional support provision) did not have a significant mediation effect. Third, emotional support reception and provision sequentially mediated the relationships between group interaction with purpose in life, life satisfaction, and personal growth. The results highlight the salient role of providing emotional support compared to receiving it, and they indicate that it is sequential reciprocity instead of equitable reciprocity that explains how group interaction benefits individuals’ psychological well-being in mutual aid contexts.

Derived from the helper theory principle, the first set of hypotheses posited a potential mediating role of emotional support provision, which was supported by the significant mediation found in the current study. Specifically, group interaction was positively associated with emotional support provision, which itself was associated with purpose in life, personal growth, and life satisfaction. However, emotional support reception did not play such a role between group interaction and psychological well-being variables, suggesting that solely receiving support does not ensure positive psychological outcomes, further supporting the helper principle [56]. The significant mediating role of emotional support provision corroborates empirical evidence in previous literature, which emphasizes an undeniable positive role of emotional support provision in enhancing psychological well-being [36,57]. In particular, the helper therapy principle explains that being a helper enhances one’s psychological well-being by improving one’s self-image, distracting attention from one’s own problems, providing status in the mutual aid group, receiving support as a reward [29,33], and, most importantly, mitigating the negative effects of receiving support by reducing the feeling of indebtedness [58]. In summary, the act of helping others is therapeutic, and providers benefit more in terms of mental well-being than the help recipients themselves [59]. Therefore, providing emotional support is a necessary component that, together with receiving emotional support, constitutes a completed support exchange process.

Equitable reciprocity, as maintained in equity theory, suggests that young PCHC would benefit most from a balanced relationship between giving and taking. To test the role of equitable reciprocity, the second set of hypotheses posited a mediation by the interaction term of emotional support reception and emotional support provision. The current study did not find any significant mediation effects of this interaction term, thus refuting these hypotheses. Specifically, engaging in equitable reciprocity did not increase purpose in life, personal growth, or life satisfaction. Past research examining equity theory has yielded mixed results: while some studies have demonstrated a significant interaction effect as evidence supporting equivalent support exchanges [32,40,58], inconclusive results have also been seen in other studies [60]. For illustration, in Pandit and Nakagawa’s (2021) [37] study, giving and receiving emotional support at high levels can alleviate depression, but this conclusion did not hold for those providing and receiving at a low level, despite it being an equitable exchange. Therefore, equity theory (which focuses on equitable exchanges) may oversimplify the reciprocal relationship of receiving and providing support [61], and the underlying mechanism seems to be more complicated than its propositions.

Based on the synthesis of the norm of reciprocity and the concept of communal relationships, the last set of hypotheses posited that emotional support reception and emotional support provision sequentially mediate the relationships between group interaction and the three aspects of psychological well-being. These hypotheses were supported by the results. Specifically, group interaction first induced emotional support reception, which triggered emotional support provision, which subsequently led to enhancement of psychological well-being, including purpose in life, life satisfaction, and personal growth. In the first place, sequential reciprocity between receiving and providing emotional support aligns with the norm of reciprocity, in which recipients have a moral obligation to return help to those who helped them [42]. Moreover, empirical evidence suggests a potential sequential relationship between receiving emotional support and providing emotional support—that is, a history of receiving emotional support from others can predict one’s behavior of providing support in the future [51]. This aligns with the current findings that receiving emotional support (baseline) led to subsequently providing emotional support (follow-up).

One possible explanation for the sequential mediating role of emotional support may be related to the nature of the mutual aid group, which can be regarded as a system of communal relationships, and the unique feature of Chinese culture. Communal relationships are generalized and flexible reciprocal relationships, wherein exchanges can occur among group members (instead of specific pairs) and can transpire in various ways in terms of how and when one repays the support one receives [62]. In Chinese societies, where the current study was conducted, communal relationships permeate most social support networks [62]. Instead of the individual-oriented and somewhat equity-driven social networks found in Western cultures, researchers have asserted that Chinese social networks are less calculative and are usually guided by mutual sharing and interdependence. Due to the influence of Confucianism, Chinese people typically strive for communal harmony and instinctively favor reciprocal relationships. Within a harmonious community, individuals who receive help will offer help to any other members in the future. The community acquires the credit and serves as a center to gather and exchange supportive resources. In traditional Chinese culture, people feel that it is obligatory to repay favors, but they do not demand the other party to repay immediately or return an equivalent amount of favor. In addition, altruism and generosity are favorably-viewed and frequently-observed traits in Chinese people [63], which often leads to proactive provision of support. Therefore, it is not surprising that emotional support reception serves as an antecedent that stimulates general emotional support provision later, without expecting anything in return. Through this type of reciprocity, participants are able to improve their ability to identify their life meaning, to achieve continuous self-development, and to increase their life satisfaction.

While sequential reciprocity may dominate in Chinese culture, equitable reciprocity is often observed in Western cultures [37]. The discrepancies merely reflect how culture may affect people’s mindsets about returning favors in mutual aid groups. Furthermore, wide cultural diversities exist that go beyond the East–West divide mentioned above. For instance, Hamilton and Sandelowski (2003) [61] demonstrated that reciprocal exchange among African Americans did not follow equity theory; instead, they have a communal orientation to reciprocity, such as repaying a third party in the future after receiving support from someone else. This is quite similar to what we depict as sequential reciprocity. Accordingly, our study contributes to the literature by providing empirical evidence for the applicability of the sequential reciprocity model to those cultural contexts with a communal reciprocity orientation.

Additionally, the current study finds that in the path model, the direct effects of group interaction on purpose in life, personal growth, and life satisfaction were non-significant. One possibility is that these non-significant direct effects may arise from their redundancy to those of emotional support reception and provision; that is, the contribution of group interaction depends on mediation by emotional support reception and provision. In other words, when group interaction does not generate emotional support reception and provision, or when the support received and provided remains constant, group interaction alone does not enhance psychological well-being. Reciprocal emotional support is necessary to establishing close and supportive bonding and to ensure authentic communications, which further leads to improvement in psychological well-being.

Finally, this study’s results suggest that different chronic health conditions have different associations with the psychological well-being of young PCHC. Despite the positive effects of emotional support provision and sequential reciprocity, asthma, diabetes, hematologic diseases, eczema, and mental illnesses still have a negative impact on psychological well-being. Individuals with chronic mental illness also suffer from symptoms that may interfere with their ability to perform daily activities such as participating in work, school, and interpersonal relationships [64]. Thus, it is not surprising that mental illnesses led to lower levels of personal growth. For other physically limiting chronic illnesses, previous studies have found empirical evidence that emotional problems such as depression and anxiety were likely to be associated with these chronic health conditions due to the change in lifestyle and illness-related distress [65,66]. For instance, diabetes and hematologic diseases require intensive self-management behaviors, such as tracking blood glucose or hemoglobin levels; managing these behaviors can be physically and emotionally draining, thus causing prolonged stress. Asthma is a potentially life-threatening disease and is likely to increase one’s anxiety and depression, which in turn can trigger more asthma attacks. Similarly, eczema causes worries about body image and triggers negative feelings due to the annoying symptoms of the condition, which can in turn lead to more eczema flare-ups. Such vicious cycles may reinforce the negative impacts on one’s life satisfaction, thus hindering one’s motivation to pursue a better life.

## 5. Contributions and Limitations

This study has both theoretical and practical contributions. Theoretically, this study revealed the dynamic process of emotional support exchanges in improving the various aspects of psychological well-being of young PCHC, and extended the understanding about how cultural variation may affect the links between group interaction and psychological well-being. In practical terms, this study’s findings have implications for facilitators in mutual aid group practices, typically social work practitioners. Given the significant benefits of sequential reciprocity in mutual aid groups, the study also offers several directions for mutual aid group practice. To start, mutual aid group facilitators should focus on promoting group interactions in order to maximize opportunities for group participants to provide support among themselves. Furthermore, mutual aid group facilitators should also work on fostering communal relationships within the mutual aid group—that is, reciprocal support oriented toward meeting members’ needs [29]. In doing so, they can help members see beyond simple exchange relationships limited to specific pairs. This constitutes a crucial action in ensuring a sequential reciprocity process, which prompts members who have received support to engage in general aid provision in the future. By adopting the sequential reciprocity approach, those who have been helped can transform their role into one of helpers (i.e., peer mentors) in the mutual aid groups. In this way, they can use their experiences to guide and support new members, which in turn can benefit themselves by enhancing their own psychological well-being [67].

Despite these contributions, the current study contains several limitations for future research to address. First, the results and conclusions rely on patients’ self-report measures, which can be biased by social desirability and recall biases. In future studies, independent data obtained from objective observation can cross-validate such results. Second, although the questionnaire adequately captured the reciprocal emotional support process longitudinally, it did not specifically identify the recipient of the support provided (i.e., whether the support was directly returned to the benefactor or indirectly to other members of the group). Future studies should measure whether the support was reciprocated directly or indirectly. Third, our study targeted younger patients between the ages of 12 and 45 years who have early-onset chronic health conditions. Thus, the age range of our participants is broad and includes a wide array of life stages. PCHC at different life stages (e.g., adolescents, young adults, and middle-aged adults) are likely to differ in how they engage with and respond to different forms of emotional support [68]. However, a subsample analysis was not possible in the present study due to the limited subsample sizes of those in the different age groups (e.g., 37 participants were in the 12–18 age range). Hence, future studies should explore the potential differences in emotional support reception and provision as well as psychological well-being among PCHC at various life stages. Furthermore, the current study involved a sample of PCHC in a Chinese society; future studies focusing on other patient groups from diverse cultural backgrounds should be conducted in order to be able to generalize the conclusions made here. Another issue worth noting is the small improvements in psychological well-being variables from baseline to follow-up. The small improvements may be due to two possibilities: First, the relatively short time interval (12 months) of the current study; second, psychological well-being at baseline was collected after 6 months (at least) of participation in mutual aid groups, thus individuals may have already improved their well-being up to that point and the current analyses are picking up on any additional change over the next year. In previous studies, researchers asserted that the positive impact of mutual aid groups can be long-lasting, but that it takes time to develop because group members need time to establish trust and mutual aid [28,69]. To enhance the clinical relevance of the present findings, future research may set the baseline at the beginning of mutual aid groups and use a longer time to investigate the underlying mechanism of how mutual aid groups function to facilitate their members’ psychological well-being. Moreover, instrumental support and informational support may also have positive impact on the psychological well-being of young PCHC, in addition to emotional support. Thus, in future research, we can include measurements about the other two types of support and evaluate their influences along with emotional support during mutual aid groups. Additionally, given the practical feasibility issue, we collected and used two waves of data instead of four. That is, data on group interaction and emotional support reception were collected at baseline, while data on emotional support provision and psychological well-being outcomes were collected at follow-up. Future studies may collect more waves of data in order to perfectly capture the sequential changes posited in our study, as well as collecting two waves of emotional support reception and provision data concurrently. Taken together, the results should be interpreted with caution due to the above limitations in data collection and analyses.

## 6. Conclusions

The current study provided empirical evidence supporting the influence and underlying mechanism of group interaction on young PCHC’s psychological well-being, and it identified the sequential mediation effects of emotional support—specifically, the sequential path from emotional support reception to emotional support provision. Only when young PCHC engage in actions of authentic emotional support and take on the “helper” role after receiving help can they improve their psychological well-being and attain long-lasting, positive outcomes of rehabilitation.

## Figures and Tables

**Table 1:** Demographic information of participants (*n* = 391).

Characteristic		%
Gender		
Female		55.1
Male		44.9
Monthly family income		
HKD 4999 (USD 644) or below		7.7
HKD 5000–9999 (USD 645–1289)		6.7
HKD 10,000–19,999 (USD 1290–2580)		13.4
HKD 20,000–29,999 (USD 2581–3870)		22.2
HKD 30,000–39,999 (USD 3871–5160)		25.9
HKD 40,000–49,999 (USD 5161–6450)		12.3
HKD 50,000 (USD 6451) or above		11.8
Educational level		
Primary (grade 6) or below		3.5
Secondary (grades 7–12)		40.9
Higher diploma or associate degree		14
Bachelor’s degree		37.0
Master’s degree or above		3.9
Chronic health condition		
Asthma		5.0
Heart disease		3.1
Diabetes		5.5
Rheumatic diseases		17.5
Neurological diseases		10.7
Hematologic diseases		6.5
Cancer		5.2
Rare diseases		4.7
Eczema		18.8
Mental illnesses		23.0
**Characteristic**	**Mean**	**SD**
Age (years)	30.2	7.7
Frequency of activities in the past six months (times)	1.8	3.7
Activity duration (hours)	1.5	2.5
Members (persons)	13.2	8.0
Core members (persons)	5.0	3.9
Number of professional facilitators (persons)	1.6	2.0
Number of nonprofessional facilitators (persons)	0.9	1.9

Note. Core members refer to members with leadership or coordination roles in the group.

**Table 2:** Descriptive statistics and bivariate correlations of key variables.

Variables	Mean	SD	1	2	3	4	5	6	7	8	9
1. GI (baseline)	34.435	34.619	1.000								
2. ESP (follow-up)	30.925	32.926	0.696 ***	1.000							
3. ESR (baseline)	30.985	33.010	0.846 ***	0.718 ***	1.000						
4. PIL (follow-up)	56.730	21.427	0.217 ***	0.378 ***	0.265 **	1.000					
5. PG (follow-up)	60.026	19.975	0.211 ***	0.387 ***	0.269 **	0.819 ***	1.000				
6. LS (follow-up)	55.158	21.535	0.213 ***	0.344 ***	0.258 **	0.758 ***	0.732 ***	1.000			
7. PIL (baseline)	56.014	21.068	0.168 **	0.219 ***	0.269 **	0.563 ***	0.503 ***	0.516 ***	1.000		
8. PG (baseline)	59.143	19.881	0.209 ***	0.202 ***	0.274 **	0.495 ***	0.578 ***	0.471 ***	0.779 ***	1.000	
9. LS (baseline)	55.849	19.729	0.247 ***	0.212 ***	0.281 **	0.478 ***	0.484 ***	0.555 ***	0.777 ***	0.747 ***	1.000

Note. GI (baseline) = group interaction (baseline); ESR (baseline) = emotional support reception (baseline); ESP (follow-up) = emotional support provision (follow-up); PIL (baseline) = purpose in life (baseline); PIL (follow-up) = purpose in life (follow-up); PG (follow-up) = personal growth (follow-up); PG (baseline) = personal growth (baseline); LS (follow-up) = life-satisfaction (follow-up); LS (baseline) = life-satisfaction (baseline). ** *p* < 0.01. *** *p* < 0.001.

**Table 3:** Absolute fit indices of the three tested models.

Models	*χ* ^2^	*df*	*χ*^2^/*df*	CFI	RMSEA	SRMR
The integrated model	3.432	1	3.432	0.999	0.079	0.008
The sequential reciprocity model	47.813	22	2.173	0.984	0.055	0.053
The equitable reciprocity model	27.784	2	13.892	0.984	0.182	0.019

**Table 4:** Coefficients for total, total indirect, and specific indirect effects of the path model.

Effects	Estimate
Effects from GI (Baseline) to PIL (Follow-up)	
Total	0.130 *
Total indirect	0.156 *
Specific indirect 1: GI (baseline)—ESP (follow-up)—PIL (follow-up)	0.122 **
Specific indirect 2: GI (baseline)—ESR (baseline)—PIL (follow-up)	−0.086
Specific indirect 3: GI (baseline)—ESR (baseline)—ESP (follow-up)—PIL (follow-up)	0.119 ***
Effects from GI (Baseline) to PG (Follow-up)	
Total	0.085
Total indirect	0.223 **
Specific indirect 1: GI (baseline)—ESP (follow-up)—PG (follow-up)	0.133 **
Specific indirect 2: GI (baseline)—ESR (baseline)—PG (follow-up)	−0.039
Specific indirect 3: GI (baseline)—ESR (baseline)—ESP (follow-up)—PG (follow-up)	0.129 ***
Effects from GI (Baseline) to LS (Follow-up)	
Total	0.100 *
Total indirect	0.183 **
Specific indirect 1: GI (baseline)—ESP (follow-up)—LS (follow-up)	0.100 **
Specific indirect 2: GI (baseline)—ESR (baseline)—LS (follow-up)	−0.015
Specific indirect 3: GI (baseline)—ESR (baseline)—ESP (follow-up)—LS (follow-up)	0.097 **

Note. GI (baseline) = group interaction (baseline); ESR (baseline) = emotional support reception (baseline); ESP (follow-up) = emotional support provision (follow-up); PIL (follow-up) = purpose in life (follow-up); PG (follow-up) = personal growth (follow-up); LS (follow-up) = life-satisfaction (follow-up). * *p* < 0.05. ** *p* < 0.01. *** *p* < 0.001.

**Table 5:** Standardized coefficients for direct effects of the path model.

Predictors	Mediators		Dependent Variables		
	ESR (Baseline)	ESP (Follow-Up)	PIL (Follow-Up)	PG (Follow-Up)	LS (Follow-Up)
	*β*	*β*	*β*	*β*	*β*
GI (baseline)	0.780 ***	0.337 ***	−0.026	−0.137	−0.083
ESP (follow-up)			0.363 ***	0.394 ***	0.297 ***
ESR (baseline)		0.421 ***	−0.110	−0.050	0.019
PIL (baseline)	0.148 **	0.124 *	0.382 ***	0.029	0.147 *
PG (baseline)	0.001	−0.008	0.129	0.483 ***	0.064
LS (baseline)	−0.028	−0.072	0.031	0.030	0.347 ***
Age	0.008	−0.013	0.041	0.017	−0.024
Gender	−0.007	0.001	−0.014	0.002	0.017
Education	−0.032	−0.069	0.084	0.010	0.018
Monthly income	−0.057	0.057	−0.055	0.013	0.039
Asthma	−0.018	−0.059	−0.125 *	−0.147	−0.097 *
Heart disease	0.014	0.005	0.019	−0.003	0.057
Diabetes	0.033	−0.078	−0.118 *	−0.090	−0.102
Rheumatic diseases	0.018	−0.011	−0.071	0.013	−0.071
Neurological diseases	−0.006	−0.017	−0.047	−0.029	−0.063
Hematologic diseases	0.004	−0.075 *	−0.052	−0.064	−0.089 *
Cancer	0.045	0.003	−0.044	−0.065	−0.080
Rare diseases	0.043	−0.082	0.012	0.043	−0.027
Eczema	0.010	−0.079	−0.085	−0.123 *	−0.171 **
Mental illnesses	0.060	−0.052	−0.072	−0.114 *	−0.107
*R* ^2^	0.730 ***	0.559 ***	0.417 ***	0.470 ***	0.437 ***

Note. GI (baseline) = group interaction (baseline); ESR (baseline) = emotional support reception (baseline); ESP (follow-up) = emotional support provision (follow-up); ESR × ESP = emotional support reception (baseline) × emotional support provision (follow-up); PIL (baseline) = purpose in life (baseline); PIL (follow-up) = purpose in life (follow-up); PG (baseline) = personal growth (baseline); PG (follow-up) = personal growth (follow-up); LS (baseline) = life-satisfaction (baseline); LS (follow-up) = life-satisfaction (follow-up). * *p* < 0.05. ** *p* < 0.01. *** *p* < 0.001.

## Data Availability

The datasets either generated, analyzed, or both, during the current study are not publicly available due to datasets containing information that could compromise the privacy of research participants. The data that support the findings of this study are available from the corresponding author (S.S.-y.N.) upon reasonable request.
